# Assessment of Breastfeeding and Weaning Practices and Their Effects on Infant Growth in Western Maharashtra: A Cross-Sectional Study

**DOI:** 10.7759/cureus.70030

**Published:** 2024-09-23

**Authors:** Josephine S Dalbhanjan, Yugantara Kadam

**Affiliations:** 1 Community and Family Medicine, Krishna Institute of Medical Sciences, Karad, IND; 2 Preventive and Social Medicine (PSM), Krishna Vishwa Vidyapeeth (Deemed to be University), Karad, IND; 3 Family Medicine, Krishna Institute of Medical Sciences, Karad, IND

**Keywords:** breast milk, early nutrition, growth assessment, protein energy malnutrition (pem), weaning food

## Abstract

Introduction: Breastfeeding provides nutrients and antibodies crucial for developing infants and their immune systems. Understanding the current breastfeeding and weaning practices helps identify areas requiring improvements to boost infant health outcomes. The World Health Organization and other health bodies recommend that babies be exclusively breastfed for the first six months of life. Then, complementary feeding should be introduced along with breastfeeding, which should be continued for up to two years or more.

Objective: This study aims to analyze the perception and practices of breastfeeding and weaning among mothers with 9- to 12-month-old infants.

Methodology: This cross-sectional analytical study was conducted at Krishna Hospital and Research Centre, Karad, for a period of six months. A validated questionnaire developed by investigators was used as the study tool. Mothers visiting the immunization clinics with their 9- to 12-month-old infants were enrolled in the study. Infants diagnosed as having inborn metabolic disorders, diabetes, heart diseases, and congenital malformations and those who were on special feeds or diets were not included in the study.

Results: The majority of the study participants (28 [33%]) initiated weaning when the infants were six to seven months of age, with the earliest being five months (8 [10%]). Five (6%) mothers could not initiate weaning until the infants were 12 months old. The most common weaning food was dal and rice with ghee (24 [29%]). A total of 50 (60%) participants used plastic bottles for feeding, and among them, 30 (36%) mothers used bottles to give water to their infants. A total of 23 (27%) participants did not feed their infants colostrum. The most common hunger cue was crying (82 [98%]), whereas the least common cue was mouth opening (8 [9%]). The incorrect practices followed by the mothers were late initiation of breastfeeding because of which colostrum could not be fed to the neonate; incorrect breastfeeding technique, that is, feeding little milk from each side of the breast without emptying one breast completely; late or no initiation of weaning foods; and offering less amount of weaning food than that required by the infant according to its nutritional demand.

Conclusion: Initiating breastfeeding early and continuing breastfeeds along with complimentary feeds are essential for optimal infant growth.

## Introduction

The World Health Organization and other health bodies recommend that neonates should be exclusively breastfed for the first six months of life. Then, complementary feeding should be introduced along with breastfeeding and continued for up to two years or more [1]. Breastfeeding, weaning, and the quality of food provided during weaning exert substantial effects on the lifelong eating pattern and health status of a child. Breastfeeding exclusively for the first six months of life ensures that the infant develops good immunity, which is crucial in the early years during which the immune system is not well-developed [2]. After the first six months, children must be provided complementary food to meet their increasing nutritional requirements, which is necessary for their proper growth and development. This process is defined as weaning. Mothers who perform weaning must be well aware of the timing of weaning, types of complementary food they can provide, and other aspects of the nutrition and health of babies [3,4].

According to a meta-analysis conducted in Tamil Nadu that investigated breastfeeding, weaning, and complementary feeding practices, the majority of the participants (81 [96.7%]) had performed exclusive breastfeeding, i.e., exclusive breastfeeding for the first six months of life. In total, 53 (63.5%) participants had initiated weaning when the infants were 6-12 months of age, and 31 (37.4%) mothers had started breastfeeding within 2-3 hours after giving birth [3]. Another similar study conducted in southern India highlights the significance of the timely introduction of nutrient-dense complementary foods [5]. Rani et al. investigated the weaning practices and nutritional status of children in a rural area of Haryana and discussed the importance of giving balanced and nutritious weaning foods to children [6].

The present study investigated the current perception and practices of breastfeeding and weaning and their effects on infant growth. The findings of this study on breastfeeding and weaning practices provide crucial insights for understanding and improving infant health and development. These findings can influence public health policies, educational programs, and healthcare practices, thereby leading to healthier populations and more efficient utilization of healthcare resources.

## Materials and methods

This cross-sectional analytical study was conducted at Krishna Hospital and Research Centre, Karad, Maharashtra, for a period of six months. Mothers visiting the immunization clinic with their 9- to 12-month-old infants were included as the study participants. A validated questionnaire, developed by investigators by referring to previous publications and consulting experts (i.e., pediatricians and gynecologists), was used as the study tool. The questionnaire was initially distributed to professors of the community medicine department, postgraduate residents, and interns to verify if the questions were direct and the language used was understandable. The questionnaire was then distributed to the mothers of the infants who visited the pediatric department for immunization of their child. If they had any difficulty with the questions, they were addressed immediately by the principal investigator. The modified B.G. Prasad scale was used to determine the socioeconomic level of the participants based on their total family income and total number of family members.

Inclusion criteria

The study only included women having children between 9 and 12 months of age.

Exclusion criteria

Mothers of infants diagnosed as having inborn metabolic disorders, diabetes, heart diseases, and congenital malformations and of those who were on special feeds or diets were not included in the study.

Sample size

Considering that 70% of the participating mothers are properly practicing breastfeeding and weaning [7], the sample size of the study was calculated as follows:

N = Z2pq/L2 L = 10% (precision) = (4 × 70 × 30)/102 = 84

The consecutive sampling technique was used to select mothers with infants. Before the commencement of the study, ethical clearance was obtained from the Institutional Ethical Committee of Krishna Institute of Medical Sciences KIMSDU/IEC/03/2023 (deemed to be university; protocol number 553/2022-2023). All study participants provided written informed consent. The privacy of the participants was maintained, and the data were kept securely with the principal investigator. Hard copies of the questionnaire were printed to collect the data, and based on the responses provided by the participating mothers, the principal investigator filled out the questionnaire.

Data collected were entered in a Microsoft Excel sheet (Microsoft® Corp., Redmond, WA) and summarized and analyzed using the Statistical Package for Social Sciences (SPSS version 28; IBM Corp., Armonk, NY).

## Results

In total, 84 mothers (age: 19-35 years; mean ± standard deviation: 26 ± 4.26 years) participated in this study. Most participants (37 [44%]) were in the age group of 25-30 years. Most participants (46 [55%]) had the socioeconomic status of class II (according to the BG Prasad scale), with 19 (35%) participants having class I socioeconomic status and 3 (3%) having class IV status. All participants (84 [100%]) were literate, with the minimum years of education being five and the maximum being 23 years. Most participants were working professionals (service; 56 [67%]). The most common type of family was nuclear (35 [42%]), whereas the least common type was a joint family having three generations living together (15 [18%]).

Sociodemographic factors were the factors investigated to determine the association with the correct practice score. The maximum obtainable score was 33. The score was later segregated into three categories: <15, 15-20, and >20. Scores less than 15 were considered poor, whereas those > 20 were considered good practice scores. The aforementioned table indicates a significant association of the scores with reproductive age, years of learning, and occupation. The type of family and socioeconomic class exhibited no significant association with the correct practice score (Table 1).

**Table 1:** Association between sociodemographic factors and practice score The socio-economic status of the participants was calculated using a modified B.G. Prasad scale

Socio-demographic factors	Practice score N (%)			Total	Significance	
	<15	15–20	>20		ꭓ^2^	p-value
Reproductive age						
19–26 years	10(17.5)	23(40.4)	24(42.1)	57(100)	ꭓ^2^=8.168	p=0.017
26–35 years	0	8(29.6)	19(70.4)	27(100)		
Years of learning						
5–15 years	8(15.4)	24(46.2)	20(38.5)	52(100)	ꭓ^2^=8.873	p=0.012
15–23 years	2(6.3)	7(21.9)	23(71.9)	32(100)		
Socio-economic class						
Class I & II	3(8.6)	9(25.7)	23(65.7)	35(100)	ꭓ^2^=5.068	p=0.079
Class III & IV	7(14.3)	22(44.9)	20(40.8)	49(100)		
Occupation						
Housewife	7(28)	8(32)	10(40)	25(100)	ꭓ^2^=8.84	p=0.012
In service	3(5.1)	23(39)	33(55.9)	59(100)		
Type of family						
Nuclear	5(14.3)	14(40)	16(45.7)	35(100)	ꭓ^2^=1.132	p=0.889
Joint	4(11.8)	12(35.3)	18(52.9)	34(100)		
Three generation	1(6.7)	5(33.3)	9(60)	15(100)		

Among the infants evaluated, 5 (6%) had severe malnutrition, 22 (26%) had moderate acute malnutrition, and 57 (68%) did not exhibit malnutrition. The incorrect practices followed by the mothers were late initiation of breastfeeding because of which colostrum could not be fed to the neonate; incorrect breastfeeding technique (i.e., feeding little milk from each side of the breast without emptying one breast completely); late or no initiation of weaning foods; and offering less amount of weaning food than that required by the infant according to its nutritional demand (Table 2). The majority of the participants (28 [33%]) initiated weaning when their infants were six to seven months of age, with the earliest being five months (8 [10%]). In total, 5 (6%) mothers could not initiate weaning until the infants were 12 months old. The most common weaning food was dal and rice with ghee (24 [29%]). In total, 41 (49%) participants used pacifiers or teethers, and 18 (21%) of them used Jeshthamadh sticks (liquorice root) as teethers. A total of 50 (60%) participants used plastic bottles for feeding, and 30 (36%) of them used bottles to give water to their infants. A total of 23 (27%) participants did not feed colostrum to their babies. The most common hunger cue was crying (82 [98%]), whereas the least common cue was mouth opening (8 [9%]) (Table 3).

**Table 2:** Association between the practice score and nutritional assessment determined using an age independent parameter of mid-arm circumference MUAC: mid-arm circumference; is an age-independent parameter for assessing the nutritional status of the child under 5 years of age

Nutritional status acquired by MUAC	Correct practice score N(%)			Total	Significance		
	<15	15-20	>20				
Severe malnutrition (<12.5)	1(20)	4(80)	0	5(100)	ꭓ^2 ^= 33.64	df = 4	p = 0.000
Moderate malnutrition (12.5-13.5)	8(36.4)	11(50)	3(13.6)	22(100)			
No malnutrition (>13.5)	1(1.8)	16(28.1)	40(70.2)	57(100)			
Total	10(11.9)	31(36.9)	43(51.2)	84(100)			

**Table 3:** Breastfeeding practices as seen in the participants during study Gutti is a traditional regional paste prepared by grinding dried fruits and herbal roots with a few drops of mother's milk

Practices of breastfeeding	N (%)	
Time of initiation of breastfeeding		
1 hour to 1 day		49(58)
2-3 day		29(35)
>4 days		4(5)
Lactation failure		2(2)
Colostrum given		
Yes		57(68)
No		27(32)
Pre-lacteal feeds given		
Honey		6(7)
Honey water		10(12)
Glucose water		2(2)
Formula		11(13)
Animal milk		30(38)
Breastfed the child by		
Completely emptying one side then giving the other side		44(52)
Fed little on each side		40(48)
Breastfeeding done on		
Demand		70(83)
Schedule		14(17)
Time of initiation of complementary feeds		
>6 months		16(19)
6–8 months		63(75)
Not initiated		5(6)
Exclusive breastfeeding done		
Yes		22(26)
No		62(74)
Reason		
Animal milk		30(38)
Formula		11(13)
Gripe water		30(38)
Gutti		59(70)
Frequency of complementary feeds		
Once a day		2(2)
Twice a day		22(26)
Thrice a day		57(68)

As shown in Table 4, the common practices observed in malnourished children were late initiation of breastfeeding, absence of colostrum feeding, and use of dal water and rice water as weaning food. The traditional practice of feeding gutti was initiated at three months of age.

**Table 4:** Wrong practices observed during the study Gutti is a traditional paste prepared by grinding dried fruits and herbal roots with a few drops of mother's milk

Wrong practices seen during the study		N (%)
Late initiation of breastfeeds		35(41)
Colostrum not given		27(32)
Pre-lacteal feeds		
Honey		6(7)
Honey water		10(12)
Gutti		59(70)
Gripe		30(38)
Usage of pacifiers and teether		41(49)
Bottle feeding		30(36)
Dal water/Rice water		22(26)

Years of education were significantly associated with the correct practice score (Table 5), underscoring the role of higher education in determining the use of correct weaning practices by mothers, which leads to appropriate infant growth. Compared with the mother’s age, per capita income, and occupation, the cultural practices followed by the mothers and those followed by their family and society that make mothers follow the set traditions play a major role in the weaning practices.

**Table 5:** Multivariate logistic regression analysis of the correct practice score and its influencing factors

Factors	B	SE	Wald	df	Sig.	Exp(B)	95% C.I. for Exp(B)	
							Lower	Upper
Age of mother	0.699	0.578	1.463	1	0.226	2.012	0.648	6.242
Years of learning	1.177	0.594	3.917	1	0.048	3.243	1.011	10.399
Income per capita	0.398	0.574	0.482	1	0.488	1.49	0.483	4.589
Occupation	0.382	0.608	0.394	1	0.53	1.465	0.445	4.825
Constant	−0.878	0.475	3.415	1	0.065	0.416	-	-

## Discussion

This study investigates various weaning practices and common nutritional behaviors among mothers, thereby highlighting significant discrepancies from the recommended guidelines on breastfeeding and weaning practices. The age group of the majority of participating women (25-30 years) and their socioeconomic status (class II) reflect a relatively literate (100% literacy) demographic that is still prone to traditional and potentially harmful practices.

Key findings

Weaning Practices

Timing of weaning: Overall, 33% of mothers initiated weaning when the infants were six to seven months of age, with the earliest being five months (8 [10%]), whereas some delayed it until the infants were 12 months of age (5 [6%]). This delay is often a result of infants’ refusal to eat weaning foods, which indicates a critical gap in education about the gradual introduction and persistence in offering varied foods for weaning. Dal and rice with ghee (24 [29%]) were the predominant weaning foods. This choice reflects reliance on traditional, easily digestible foods but suggests a lack of diversity in nutrient sources.

Use of pacifiers and feeding bottles: In total, 41 (49%) participants used pacifiers or teethers, and 18 (21%) of them used Jeshthamadh sticks (liquorice root) as teethers. While using pacifiers is common, the use of liquorice root, which reflects the influence of traditional practices, can pose health risks. A total of 50 (60%) participants used plastic bottles for feeding, and 30 (36%) of them used bottles to give water to their infants, highlighting the potential risks associated with hygiene and bottle-feeding.

Colostrum Feeding

In total, 23 (27%) participants did not feed colostrum to their infants, thereby missing out on its critical immunological benefits. This practice indicates a substantial gap in knowledge or adherence to traditional beliefs over scientific recommendations.

Prelactal Feeds

Practices such as feeding honey (10 [12%]) and gutti (59 [70%]) were prevalent, indicating deeply ingrained cultural practices. Honey feeding poses a risk of infant botulism, whereas gutti feeding presents nutritional inadequacy.

Breastfeeding Duration

Cessation of breastfeeding by seven months, largely because of mothers' employment, deviates from the recommended two years. This finding underscores the need for supportive workplace policies and education about the significance of breastfeeding for both the mother and the baby.

Hunger Cues

Crying was the most recognized hunger cue (82 [98%]), whereas mouth opening was the least recognized (8 [9%]). This disparity indicates that mothers must be educated on recognizing subtle hunger cues.

Correlation With Education

According to the multivariate logistic regression analysis, years of education significantly correlated with the correct weaning practices. As educational attainment increased, adherence to proper weaning guidelines also increased. This emphasizes the critical role of education in promoting healthy practices of infant feeding.

Comparative findings from similar studies

Urban Slums of North India

Similar to our study, a study [8] conducted in the urban slums of North India reported early initiation of gutti feeding (as early as three months) and a high prevalence of prelactal feeds, including honey water and honey from the forest, with similar misconceptions about their health benefits. This finding reiterates the notion that traditional practices are widespread and not confined to specific regions.

Rural Communities in South India

Kumar et al. highlighted that delayed weaning and the use of dal water and rice water for weaning are common practices in rural South India, and these practices were often influenced by familial and societal norms [9]. The authors also found that educational interventions significantly improved weaning practices.

Socioeconomic Influences

The study by Gupta et al. in semi-urban areas unveiled that socioeconomic status plays a crucial role in weaning practices [10]. Mothers from higher socioeconomic classes tended to follow the recommended guidelines more closely, whereas those belonging to lower socioeconomic groups adhered to traditional practices, which often led to malnutrition.

Summary

The study findings indicate a considerable need for educational interventions for mothers targeted at enhancing their awareness about the benefits of correct weaning practices and the risks associated with traditional but harmful practices. Supportive workplace policies can help prolong breastfeeding and ensure better infant nutritional outcomes. On comparing with similar studies, we observed that these issues are prevalent across different regions and socioeconomic groups, underpinning the universal need for improved maternal education and support.

Study limitations

The study sample size may not be sufficiently large to accurately represent the broader population. Consequently, the study findings might not be generalizable to the mothers in different regions or with diverse backgrounds. The study relied on self-reported data from mothers regarding their breastfeeding and weaning practices. This introduces the potential for recall bias and social desirability bias, which could have affected the accuracy and reliability of the reported information. This cross-sectional study captures practices and outcomes at a single point in time. This study design limits the ability to determine causality between breastfeeding and weaning practices and the observed nutritional outcomes in infants. The study might not have completely considered the diverse cultural and socioeconomic factors influencing breastfeeding and weaning practices. This can affect the interpretation and applicability of the findings across different settings. Variations in access to healthcare services and breastfeeding support were not thoroughly examined. Differences in support systems can significantly affect the mothers’ ability to adhere to recommended practices, thereby influencing the study outcomes. The methods used to assess the nutritional status of the infants may be limited in precision and scope, potentially overlooking other factors, apart from breastfeeding and weaning practices, that contribute to infant growth and development.

Recommendations based on study findings

The target groups with a low practice score and incorrect practices observed during this study were mothers with fewer years of education and those living in a joint family where they are not the decision-makers and are influenced by too many people around. Breast milk should be initiated within 30 minutes of delivery. The delay in initiating breastfeeding leads to an adjournment in the development of oxytocin reflexes, which are essential for the contraction of the uterus and the breast milk reflex [11]. Correct feeding practices should be followed, that is, completely emptying one side of the breast and then feeding through the other side. This practice can ensure that the infant receives both fore and hind milk. Colostrum was not fed by 27% of the participants. Colostrum is rich in vitamins, minerals, protein, and immunoglobulins that protect infants from infections [12]. Honey should be avoided in children below the age of one year as it may cause Clostridium botulinum infection, which can be fatal [13]. Dal water and rice water should be given only when the infant has diarrheal episodes and not as complementary foods. The cultural practice of feeding gutti was found to be initiated as early as three months. This practice cannot be avoided completely. However, gutti can be prepared hygienically and started as infants reach five to six months of age [14]. Working mothers from nuclear families commonly used pacifiers/teethers (49%) and bottle feeding (36%) as they could not continue breastfeeding due to work schedules. Therefore, appropriate washing of these products with baby-friendly solutions is recommended to minimize the spread of infection to infants via the oral route.

## Conclusions

This study highlights several crucial aspects of breastfeeding and weaning practices commonly employed by mothers. First, while a majority of mothers initiate breastfeeding, adherence to exclusive breastfeeding for the recommended first six months of life is significantly influenced by factors such as maternal education, socioeconomic status, and cultural beliefs. A lack of knowledge and cultural traditions often results in the early or late introduction of complementary foods, causing deviation from the recommended age of six months for introducing such foods. This improper weaning timing arises from misunderstandings about the nutritional needs of infants and the benefits of timely weaning.

Furthermore, the nutritional impact is evident from the findings of this study, as infants exclusively breastfed for the first six months of life and then introduced to appropriate complementary foods exhibited better growth metrics. By contrast, early or late weaning correlates with nutritional deficiencies or excessive weight gain. Culture substantially influences and shapes these practices, with certain beliefs and family traditions leading to early cessation of breastfeeding or inappropriate weaning practices. The study, thus, underscores the significance of proper breastfeeding and timely weaning for the optimal growth and development of infants. The findings emphasize the need for enhanced educational programs and public health interventions to improve maternal practices and promote better health outcomes for infants.

## Appendices

**Table 6:** Questionnaire asked during the study

Questionnaire					
Demographic details					
A. Serial No.					
B. Age of the mother C. Mother’s education					
D. Occupation of the mother E. Type of family					
F. Total Family Income G. Total family members					
H. Age of child in months					
(Q-1) Time of initiation of breastfeeding					
(Q-2) Colostrum feeding? (Yes)/(No)					
(Q-3) Did you feed the child anything other than breastmilk in the first 6 months?					
If yes what was given?					
a) Honey b) Honey water c) Sugar water d) Glucose water e) Other (pl specify)					
Q-4) While breastfeeding the child					
a) Did you feed the child on both side of the breast?					
b) Did you completely empty one side and then go to the other?					
c) Little on both sides					
(Q-5) Was the breastfeeding done on demand or on schedule?					
(Q-6) How do you recognize that the baby is hungry?					
a) Crying b) Irritability c) Mouth opening d) Turning head to one side					
e) Sucking thumb/fingers/hands					
(Q-7) Before breast feeding, do you wash/wipe your breasts?					
(Q-8) Do you give water to your child? If yes, since when?					
(Q-9) Do you feed gutti/gripe water to your child? If yes, since when?					
(Q-10) Do you put pacer/pacifier/teether in your baby’s mouth?					
(Q-11) At what age did your start weaning your child?					
(Q-12) What foods did you give to start weaning in your child?					
(Q-13) Do you feed eggs to your child? If yes, since when did you start feeding them?					
(Q-14) Do you give non-veg soup to your child? If yes, since when did you start giving? Do you mix anything thing else in that soup					
(Q-15) How much quantity of weaning food do you give to your child?					
a) One-fourth bowl b) Half bowl c) Small bowl					
(Q-16) How many times in a day do you feed weaning foods to your child?					
(Q-17) Did you reduce breastfeeding after initiating weaning foods to your child?					
(Q-18) After introduction of weaning foods, did the child show any signs of indigestion?					
a) Vomiting b) Diarrhea c) Excessive crying due to stomach pain					
d) Refusal to eat weaning foods and demanding for milk					
(Q-19) Do you bottle feed the child? If yes, since when ?					
(Q-20) Has the child fallen sick till now? Was he admitted?					
(Q-21) When you were sick, did you feed the child?					
(Q-22) When the child was sick, did you feed the child?					
